# Aortic Vascular Graft and Endograft Infection–Patient Outcome Cannot Be Determined Based on Pre-Operative Characteristics

**DOI:** 10.3390/jcm13010269

**Published:** 2024-01-03

**Authors:** Ilaria Puttini, Marvin Kapalla, Anja Braune, Enrico Michler, Joselyn Kröger, Brigitta Lutz, Natzi Sakhalihasan, Matthias Trenner, Gabor Biro, Wolfgang Weber, Thomas Rössel, Christian Reeps, Hans-Henning Eckstein, Steffen Wolk, Christoph Knappich, Susan Notohamiprodjo, Albert Busch

**Affiliations:** 1 Department for Vascular and Endovascular Surgery, Klinikum Rechts der Isar, Technical University of Munich, 80333 Munich, Germany; 2 Division of Vascular and Endovascular Surgery, Department for Visceral, Thoracic and Vascular Surgery, Medical Faculty Carl Gustav Carus, University Hospital, Technical University of Dresden, 01307 Dresden, Germany; 3 Nuclear Medicine Department, University Hospital Carl Gustav Carus, Technical University of Dresden, 01307 Dresden, Germany; 4 Department of Cardiovascular and Thoracic Surgery, University of Liège, 4000 Liège, Belgium; 5 Division of Vascular Medicine, St. Josefs-Hospital Wiesbaden, 65189 Wiesbaden, Germany; 6 Department of Nuclear Medicine, Klinikum Rechts der Isar, Technical University of Munich, 81675 Munich, Germany; 7 Department of Anaesthesiology and Critical Care Medicine, University Hospital Carl Gustav Carus Dresden, Technical University of Dresden, 01307 Dresden, Germany

**Keywords:** aortic graft infection, vascular graft endograft infection, pericardial prosthesis, silver graft

## Abstract

Vascular graft/endograft infection (VGEI) is a serious complication after aortic surgery. This study investigates VGEI and patient characteristics, PET/CT quantification before surgical or conservative management of VGEI and post-intervention outcomes in order to identify patients who might benefit from such a procedure. PET standard uptake values (SUV) were quantitatively assessed and compared to a non-VGEI cohort. The primary endpoints were in-hospital mortality and aortic reintervention-free survival at six months. Ninety-three patients (75% male, 65 ± 10 years, 82% operated) were included. The initial operation was mainly for aneurysm (67.7%: 31% EVAR, 12% TEVAR, 57% open aortic repair). Thirty-two patients presented with fistulae. PET SUV_TLR_ (target-to-liver ratio) showed 94% sensitivity and 89% specificity. Replacement included silver-coated Dacron (21.3%), pericardium (61.3%) and femoral vein (17.3%), yet the material did not influence the overall survival (*p* = 0.745). In-hospital mortality did not differ between operative and conservative treatment (19.7% vs. 17.6%, *p* = 0.84). At six months, 50% of the operated cohort survived without aortic reintervention. Short- and midterm morbidity and mortality remained high after aortic graft removal. Neither preoperative characteristics nor the material used for reconstruction influenced the overall survival, and, with limitations, both the in-hospital and midterm survival were similar between the surgically and conservatively managed patients.

## 1. Introduction

Aortic vascular graft and endograft infections (VGEIs) are among the most challenging cases in vascular surgery for both clinicians and patients. Despite the more frequent use of endovascular aortic repair for aortic occlusive disease (AOD) as well as aortic aneurysm (EVAR/TEVAR) over traditional open repair (OR), the incidence of VGEI is on the rise and estimated to affect 1.5–4% of the respective population [1,2].

Typical symptoms range from unspecific fever and pain to impaired wound healing to catastrophic bleeding events in the case of aorto-enteric fistula and might occur early or late (>4 months) after implantation [3]. In order to facilitate VGEI diagnosis, criteria based on clinical/surgical, radiologic and laboratory findings have been defined, providing excellent sensitivity (up to 99%) and high specificity (up to 61%) [2,4]. Recently, ^18^F-fluordeoxyglucose (FDG) positron emission tomography (PET) is being used more frequently and was demonstrated to increase diagnostic specificity over computed tomography angiography (CTA) [5,6]. Yet, uncertainty remains about a possible diagnostic cut-off regarding a pathologic maximum standard uptake value (SUV_max_) [3,7]. 

Treatment and specific outcomes for aortic VGEI are reported in a few retrospective cohort studies of 20–80 patients, mostly focusing on the abdominal aorta and a specific replacement material or technique after a follow-up of six months to five years [8,9,10]. Here, upon graft removal, early and late mortality have been summarized to vary between 4.3–48% and 9–85%, respectively [3,11]. Conservative treatment with or without eventual drainage, irrigation and lifelong anti-infectives is palliative and only scarcely reported [12]. If no bleeding or fistulas occurred, singular cases were shown to have good life expectancy [13,14]. In approximately one-third of cases, blood cultures or intraoperative swabs remain negative. Missing reporting standards on sample acquisition, time-to-infection, antibiotic regimen or infectious specificity make identification of the responsible (poly)microbial flora impossible [2,15]. Hence, various meta-analyses and one European guideline agree on the lack of robust evidence and the continuous need for primary data in this ever-evolving clinical problem [3,16,17].

In this dual-center retrospective cohort study, we report the clinical presentation, procedural and outcome details of VGEI patients treated operatively and conservatively. Additionally, we aim to identify a possible benefit of preoperative risk stratification regarding outcomes involving a quantitative and qualitative PET/CT analysis based on patient and diagnostic characteristics.

## 2. Patients and Methods

Study design, patient identification and ethical approval: A dual-center retrospective cohort study was conducted. Patients with VGEI were identified at two university centers retrospectively from the electronic information system, operative charts and prospective case registration for the purpose of VGEI cohort analysis from 1 January 2013 (Munich) or 2015 (Dresden) to 31 December 2021. Baseline data were retrieved from electronic patient records and follow-up visits. 

Patient data were pseudonymized for further analyses. The study was performed in accordance with the Declaration of Helsinki and approved by the local ethics committees (Medical Faculty, Technical University of Munich: 2022-428-S-NP and Technical University Dresden BO-EK-205042022). The STROBE checklist (v4) for cohort studies was followed as far as possible [18].

Inclusion/exclusion criteria: All patients operated on or diagnosed with their first thoracic or abdominal aortic VGEI were included. Diagnosis was established using MAGIC (Management of Aortic Graft Infection Collaboration) criteria positivity (s. below) from Lyons et al. [2].

Patients <18 years old and patients with isolated iliac graft infections were excluded.

*Conservative treatment group:* All patients who refused definitive operative treatment after informed consent (ideally including relatives) and patients who were considered unfit for surgery after thorough evaluation including complete cardiac and pulmonary workup and vascular board decision did not undergo graft removal or replacement but may have undergone drainage and/or irrigation. Here, all patients were dismissed on calculated (if available) or empiric (individual case discussion with infectiologist) anti-infective therapy.

Stepwise analysis and definitions: *Patient baseline characteristics* included age, sex, hypertension, diabetes, smoking status, chronic obstructive pulmonary disease (COPD), ethanol consumption, renal insufficiency (any Kidney Disease: Improving Global Outcomes [KDIGO] ≥ 2), permanent dialysis, active/previous malignancy, hyperlipidemia, coronary artery disease (CAD) and peripheral arterial occlusive disease (PAOD). 

*Initial aortic operation* was classified as EVAR (including fenestrated and scalloped grafts (n = 2) and iliac branch devices (n = 1) as well as monoiliac reconstruction in combination with extra-anatomic crossover bypass (n = 2)). Furthermore, TEVAR or open aortic repair (OAR) (including tube or aorto-bi/mono-femoral/iliac grafts) for aneurysm (or penetrating aortic ulcers (PAU)), occlusive disease or dissection was noted. Additionally, initial operation due to aortic rupture and in/ex domo procedure was recorded. 

*A Diagnostic protocol* was followed in all cases with suspicion of VGEI. This included blood cultures/swabs (fistula) before antibiotics, CT and, in case of GI bleeding or suspicion of fistula, diagnostic endoscopy. PET/CT was indicated in case of doubt after initial assessment or for confirmation in selected cases. No bacterial DNA was assessed.

*VGEI characteristics* included time-to-VGEI defined by the date difference between initial operation and established VGEI diagnosis. According to MAGIC criteria, diagnosis was established when at least a single major criterion and any other criterion from another category was fulfilled [2,3]. MAGIC criteria (radiologic) were based on the last CT and/or PET/CT or PET/MR before established diagnosis and the corresponding sampling (microbiologic) via (1) blood culture, (2) CT-guided aspiration and/or (3) direct swab (i.e., cutaneous fistula). Cut-off between late and early VGEI was >4 months [3].

Preoperative CT-guided drainage (or drain only in the conservative treatment group) was for diagnostic purposes, and fluid drainage and eventual temporary irrigation were at the surgeon’s discretion. No other procedures were carried out in conservatively managed patients.

*Operative handling and definitions:* Indication for graft replacement was made via a vascular board decision with patients’ (and ideally relatives’) informed consent. Emergency surgery was performed due to life-threatening bleeding. Urgent surgery was performed due to suspected aorta-related symptoms (n = 2 summarized with elective procedures). Gastrointestinal fistula involved esophagus and duodenum. The material and type of reconstruction was left to the surgeon’s discretion, with the goal of removing all graft material (bare springs eventually left in situ), and extensive debridement of the surrounding infectious tissue was performed as far as possible. Generally, anatomical reconstruction and primary abdominal closure were aims. Eventual additional procedures, including omentum plasty, renal cold perfusion or preemptive temporary left-heart bypass, and especially intestinal reconstruction or discontinuation, were at the treating surgeon’s discretion and shown as direct suture (no anastomosis) or resection (including anastomosis and resection).

Additional CT-guided drainage during the postoperative course was performed when necessary. Postoperative complications were aortic (bleeding, rupture), neurologic (stroke, critical illness neuropathy, procedure related nerve injury, delirium), medical (acute kidney failure (any worsening of initial KDIGO stage), temporary dialysis, respiratory problems (longtime respirator treatment, pneumonia, edema), pulmonary embolism, myocardial infarction) and surgical (surgical site infection [SSI], limb ischemia, visceral complication [intestinal ischemia, insufficiency]). Complications were additionally classified according to the Clavien–Dindo classification [19].

*Reinfection* or persistent infection of the aorta (replacement graft) or the operative situs was defined as suggested by the ESVS guidelines by either (1) any new signs of sepsis/systemic inflammatory response (SIRS) when other sources were ruled out; (2) newly diagnosed bacteria/fungi from periaortic fluid/abscess drainage; (3) newly diagnosed VGEI after replacement according to MAGIC criteria; or (4) anastomotic rupture due to infected graft [1,2,3]. Reinfection was diagnosed in-hospital or during follow-up and is summarized in results.

*Additional complications during follow-up* were aortic/graft-related complications (anastomotic rupture; high-grade limb stenosis) requiring reintervention (i.e., secondary extra-anatomic reconstruction, endovascular lining), other surgical complications requiring operation (ureter stenosis, SSI, limb or mesenteric ischemia) and medical complications remotely related to the previous replacement (kidney failure, subileus, pulmonary embolism). Patients in the conservative treatment group were not followed up for complications. All patients or their family physician were contacted in 2022 to establish last contact if not visible from electronic records. 

Endpoints and outcome parameters: Primary endpoints were in-hospital mortality (safety endpoint) and 6-month aortic reintervention-free survival (efficacy endpoint).

Secondary endpoints were mortality (at 30 d, 6, 12 months and overall) and the in-hospital complication rates (aortic, surgical, medical, neurologic).

Additionally, the aortic/surgical complication rates and the overall survival in relation to the graft material for aortic reconstruction were analyzed. 

PET image acquisition and quantitative and qualitative analysis:

*^18^F-FDG-PET acquisition:* All PET/CT examinations were performed on a Biograph-Vision 600, a Biograph mCT machine, or a Biograph16 Hirez (Siemens Healthineers, Erlangen, Germany). Two patients received a PET/MR examination using a 3 Tesla Ingenuity TOF PET/MRI (Philips Medical Systems, Best, The Netherlands). 

Patients fasted for at least 4 h prior to the ^18^F-FDG tracer injection. Blood glucose levels were required to be less than 140 mg/dL during a period of approximately 60 min before the administration of the ^18^F-FDG. Diagnostic CT imaging was performed in the portal venous phase 80 s after intravenous injection of contrast agent [Imeron 300] (1.5 mL/kg body weight, max. 120 mL) followed by the PET imaging in continuous bed motion mode (“flow-mode”) or vice versa. 

PET image acquisition techniques differed slightly between the two centers involved and the three different machines applied over time. Generally (Dresden cohort), a median of 4,73 MBq/kg body weight ^18^F-FDG (range: 2.88–6.39 MBq/kg) was intravenously injected, and PET image acquisition started after a median of 76 min p.i. (range: 58–113 min). Images were acquired in continuous bed motion mode (Biograph mCT: 1.5 mm/s; Biograph Vision 600; 2.2 mm/s) or in step-and-shoot mode (Biograph16 Hirez PET/CT: 2 to 3 min per bed position, Ingenuity PET/MRI: 2 min per bed position).

All PET/CT examinations were reconstructed using an ordered subset expectation maximization (ODEM) iterative reconstruction algorithm and were corrected for randoms, dead time, scatter and attenuation (Biograph mCT: time-of-flight measurements, 3D reconstruction using point-spread-function modeling, three iterations, twenty-one subsets, 200 × 200 matrix, zoom 1.0, defined voxel size, post-reconstruction smoothing (Gaussian filter) or were acquired in 3D mode with an acquisition time of 1.5 mm/s from 2019.

*Image Analysis:* All ^18^F-FDG-PET scans were analyzed with syngo.via (software version VB30A, Siemens Healthineers, Erlangen, Germany) under the supervision of an experienced nuclear medicine physician (SN) on a certified digital workstation who was blinded to the clinical data. Quantitative uptake was analyzed with Osirix (Osirix MD, Pixmeo, Geneva, Switzerland). The following parameters were acquired for quantification:

*Intensity uptake*: SUV_max_ was assessed for aorta, liver and mediastinal blood pool using region of interest (ROI) analysis, which was manually drawn based on co-registered CT or MR images. In all examinations, 10 cm³ circular regions of interest (ROIs) were drawn. The SUV_max_ in the region of the vascular graft was defined as SUV_max_ for the aorta. The SUV_max_ for liver and mediastinal blood pool were recorded in the 7th segment and in the supravalvular ascending aorta, respectively. Calculated values included the SUV_TLR_ (target-to-liver ratio) and the SUV_TBR_ (target-to-back ratio) for better comparison to exclude individual tracer dosage-dependent effects. (Whole body datasets were analyzed for secondary diagnosis, that is, infectious foci not in the vicinity of a graft, or other malignant findings as part of the routine diagnostic procedure). Additionally, intensity uptake was assessed using a visual grading scale (VGS: Grade 0: no uptake; Grade 1: low ^18^F-FDG uptake, lower than the mediastinal blood pool; Grade 2: moderate uptake between the mediastinal blood pool and the liver uptake; Grade 3: high uptake, moderately higher than the uptake of the liver; Grade 4: strongest uptake, markedly higher than the uptake of the liver).

*Focality uptake*: Focal FDG uptake was defined as well-circumscribed areas of increased uptake in connection with the graft. The FDG pattern was classified as “uni- or multisegmental” for focal or diffuse uptake or “circumferent or semicircumferent” for a pattern that was encircling the aortic graft entirely or partially. Additionally, a description of the localisation of the uptake (aneurysm sack, aortic wall, graft) was documented. 

*Non-VGEI control group:* For comparison, 19 patients who had ^18^F-FDG-PET/CT for other reasons (cancer) and no signs of VGEI or history of graft occlusion related to their previously implanted graft based on the above mentioned criteria were selected consecutively from an institutional study database.

Statistical analysis: Statistical analysis was performed using IBM SPSS for Windows, Version 28.0 (IBM Corp., Armonk, NY). Dichotomous variables were recorded as absolute frequencies (number of cases) and relative frequencies (percentages). Continuous data are presented as mean ± one standard deviation (SD) and non-symmetrical data with median and interquartile range (IQR). Pearson’s chi-squared or Fisher’s exact test was used to analyze the categorical variables. Differences between means were tested with the t-test or the Mann–Whitney U test. Distribution of normality was assumed using histograms where applicable. Survival and patency data were analyzed using Kaplan–Meier estimates, and differences were analyzed using the log-rank test. Univariate Cox regression was used for analysis of risk factors for aortic reinfection with hazard ratios (HR) and 95% confidence intervals (CI) as measures of association. Univariate binary logistic regression analysis was performed to evaluate risk factors for in-hospital mortality with odds ratios (OR) and 95% CI as measures of association. The calculation of cut-off values of PET parameters was determined using receiver operating characteristics (ROC) curve analysis and Youden Index. Analysis of the area under the curve (AUC) of the ROC curves was performed using Delong’s test to compare the performance of PET parameters for a predictive response. A two-sided *p*-value < 0.05 was considered statistically significant in all performed tests.

## 3. Results

### 3.1. Study Cohort

**Figure 1:**
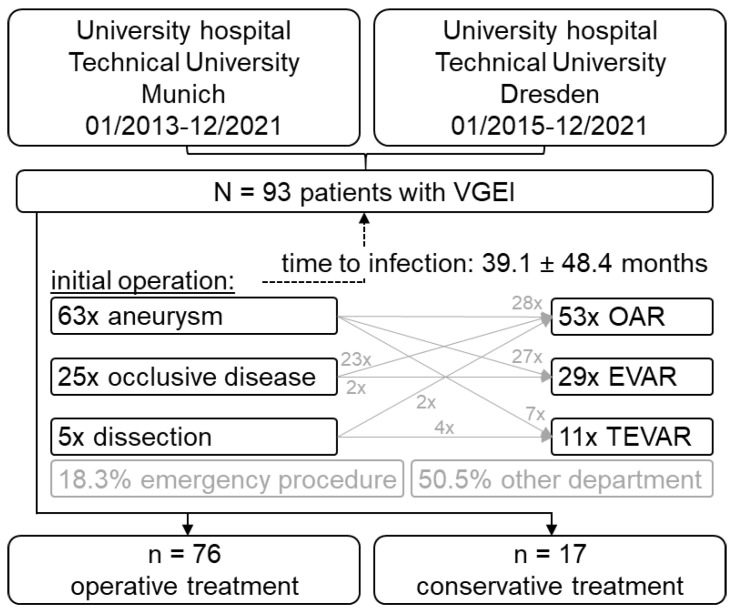
Patient flow chart.

Overall, 93 VGEI patients (24.7% female, 65.4 ± 10.6 years old) over a nine-year period were included. Of those patients, 29 (31.2%) initially had EVAR, 11 (11.8%) TEVAR and 53 (57%) OAR for aortic aneurysm, occlusive disease or dissection (Figure 1, Table 1). Detailed patient and VGEI characteristics are shown in Table 1/Supplementary Table S1.

The median interval between the initial operation and VGEI diagnosis was 24 months (95% CI: 29–49). Twenty-eight cases (30%) were early infections (Table 1). Fistulas were seen in 35.5% and unspecific B symptoms in 51.6% of the patients. Gastrointestinal fistula (n = 21) was most frequent (Table 1, Supplementary Table S1). All patients fulfilled the MAGIC criteria of VGEI, and major radiologic (CT) and laboratory criteria were met by >80% of patients (Supplement Table S2). 

Initial laboratory infectious parameters were not altered in general and microbiologic workups, which revealed 49 different bacteria/fungi with no obvious frequency patterns of species or categories in regard to the mode or localization of acquisition (Table 1/Supplementary Table S1). Preoperative blood cultures were taken in 68 patients, yet 67% proved negative. Twenty-five patients had a diagnostic puncture (8% sterile). In twelve patients, the intraoperative swab/graft sonication remained sterile (15.8%) (Supplementary Table S1). Data regarding the pre-/perioperative antibiotic regimen could not be retrieved sufficiently in order to systematically analyze the possible germ selection between infectious diagnostics.

### 3.2. Quantitative and Qualitative PET/CT Analysis

**Figure 2:**
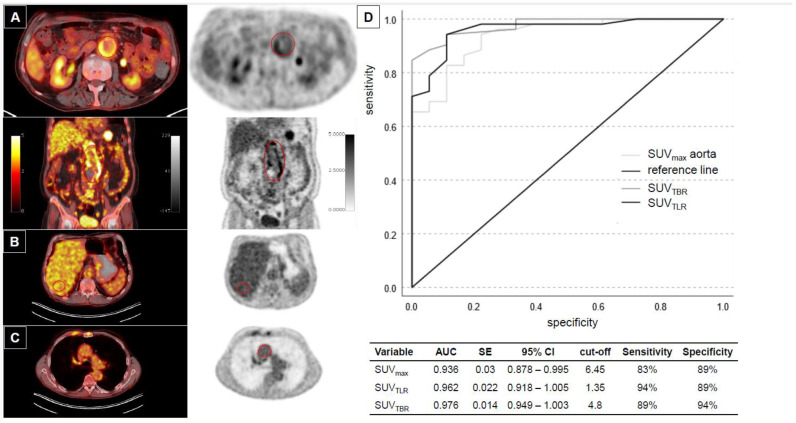
Positron emission tomography (PET)/CT and sensitivity/specificity analysis. (**A**) Axial and coronary depiction of PET/CT overlay and PET scan with correspondingly marked region of interest (ROI; red circle) where mean standard uptake value (SUV) is measured. Additional measurements are made in the liver (**B**) and the ascending aorta (mediastinal blood pool) (**C**). (**D**) Receiver-operator curve (ROC) for sensitivity/specificity based on area under the curve (AUC) measurement for SUV_max_ aorta, SUV_TBR_ and SUV_TLR_. (SE = standard error; CI = confidence interval; cut-off at maximum sensitivity and specificity).

PET/CT analysis was possible in 53 VGEI patients (60.0%) (Table 2, Figure 2A–C). Subgroup patient details and VGEI characteristics are shown in Supplementary Table S3. The mean aortic SUV_max_ was 12.5 ± 7.3, and the ratios for liver and background were 3.6 ± 2.0 (SUV_TLR_, target-to-liver SUVmax) and 4.8 ± 3.1 (SUV_TBR_, target-to-background SUVmax), respectively (Table 2, Supplementary Table S1).

A control group of 19 patients post EVAR or OAR with no clinical suspicion of VGEI was available for comparison from an institutional PET/CT cancer database (Supplementary Table S3). Here, the aortic SUVmax and the respective dependent ratios were significantly higher for VGEI patients, while liver and blood pool enrichment did not differ between the groups (Table 2). Descriptive intensity uptake based on a VGS was significantly different between patients with graft infections versus controls (*p* < 0.001) (Figure S1, Supplementary Table S1).

In a receiver-operator curve (ROC) analysis for VGEI, a SUV_TLR_ of 1.35 showed 94% sensitivity and 89% specificity (Supplementary Table S1, Figure 2D). However, SUV_max_ aorta and SUV_TBR_ performed almost equally well. Notably, all three values were higher in patients initially treated using endovascular means, whereas the time-to-VGEI and the presence of a fistula did not matter (Table 2).

### 3.3. Treatment Strategy

Across the entire cohort, 76 patients were treated operatively and 17 were managed conservatively (Table 1). Conservative treatment included long-term anti-infective treatment in all patients and CT-drainage in six patients. Conservatively managed patients were followed up at least once via telephone interview after demission. 

For operatively treated patients (all anatomical reconstruction), abdominal replacement was most frequent (84.2%), and 6.5% were emergency procedures (Table 3). The operation time was 502 ± 159 min, and a bifurcated graft (74.7%) and a physician-made pericardium graft (61.3%) were used most frequently. Additional procedures included renal cold perfusion and partial left heart bypass for distal and/or selective perfusion (described previously) [20]. Of 31 additional simultaneous procedures, intestinal resection was most frequent (Table 3).

Postoperatively, an additional CT-guided drainage was necessary in 28 cases. Most notably, aortic complications (rupture, anastomotic bleeding) were noted in 18 cases. Stroke was rare, yet surgical and medical complication rates were considerably high (61.8% and 65.9%, respectively) (Table 3). Hence, surgically treated patients spent 20 ± 37 days in the intensive care unit (ICU) and 51 ± 38 days in the hospital (Table 4).

### 3.4. Outcome Analyses

**Figure 3:**
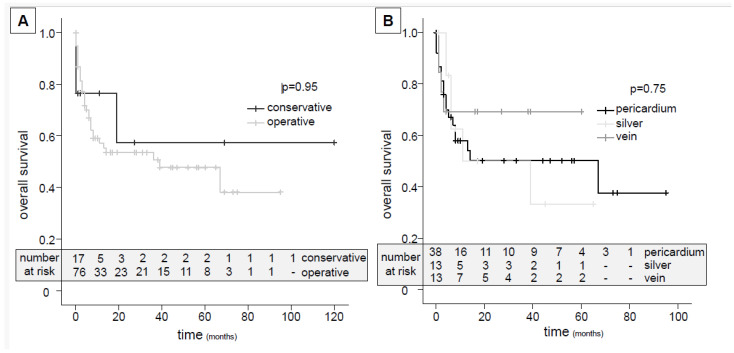
Outcome analysis. (**A**) The overall survival is shown as a Kaplan–Meyer plot comparing operative and conservative treatments for the entire vascular graft and endograft infection (VGEI) cohort (N = 93). (**B**) The overall survival is shown for the three different vascular substitutes used in the abdominal replacement group (N = 64) (log-rank test for comparison; *p* < 0.05 is considered significant).

During 22.9 ± 26.4 months of follow-up, the in-hospital mortality (safety endpoint) was 19.4% (Figure 3A, Table 4). After discharge, ten patients (10.8%) were lost to follow-up. At six months, the mortality rate was 30.1%, and the aortic reintervention free-survival rate (efficacy endpoint) was 50%. Persistent or reinfection was present in 100% of the conservatively treated patients and in 39.5% of the patients in the operative treatment group (Table 4).

While the average time in hospital exceeded one month (45 ± 37 days), the 30-day-, in-hospital-, 6-months-, 12-months and overall mortality gradually increased from 11.8% to 43% (Table 4). Here, no obvious differences were seen between operative and conservative treatments; however, the number at risk decreased rapidly (Figure 3A, Table 4). In addition to the high in-hospital complication rates, the aortic complication and reintervention rates after demission reached up to 30% (Table 4). Analyzing the materials used for abdominal replacement only (n = 64: 38× pericardium, 13× silver-coated Dacron, 13× femoral vein), no significant differences were seen regarding the reinfection or overall survival rate (Figure 3B, Table 5). Higher SUV ratios in PET did not correspond to in-hospital death (Table 2). Specific focality uptake (i.e., aneurysm sac enrichment) analysis was not associated with a specific outcome. 

**Figure 4:**
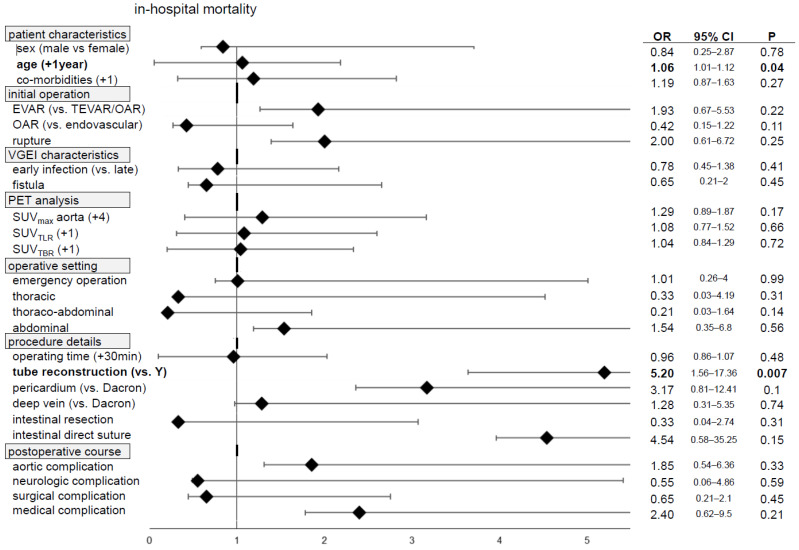
Forest plot shows selected univariate odds ratios for in-hospital mortality, with 95% confidence intervals and *p* values (logistic regression and Wald test; *p* < 0.05 is considered significant and highlighted bold; all values shown in Supplementary Table S5).

Finally, univariate analysis for in-hospital mortality revealed significantly increased odds ratios (OR) for AAA as an initial indication (OR 4.76, *p* = 0.047), B-symptoms upon clinical presentation (OR 4.22, *p* = 0.02) and tube reconstruction (OR 5.2, *p* = 0.007) (Figure 4, Supplementary Table S5). The reinfection (persistence) rate was significantly associated with emergency replacements (OR 2.41, *p* = 0.035) and mesenteric ischemia during hospital stays (OR 4.5, *p* = 0.0002) (Supplementary Figure S2, Supplementary Table S6).

## 4. Discussion

This study represents one of the largest retrospective cohort studies on VGEI and demonstrates that postoperative morbidity and mortality remain considerable high in this vulnerable patient cohort, but these results were similar to the results obtained in a limited conservative treatment group. While PET is a highly sensitive method to detect VGEI, a qualitative and quantitative approach did not correlate with the clinical endpoints.

Regarding the safety endpoint, we found an overall in-hospital mortality of 19.4%, with no differences between operative vs. conservative treatment (Figure 3A, Table 4). Similarly, the in-hospital complication rates were considerably high and did not change over time. This is in line with the literature overview provided in the 2020 ESVS guidelines, with early mortality ranging from 8–48% and graft-related (aortic) complications from 3–37.2% [3]. One additional systematic review from 2023 found an overall mortality of 14.8–27% in a short-term analysis [16]. Long-term follow-up data (>6 months) are largely missing in these patients, and if provided, the numbers at risk decrease rapidly (Figure 3A, Table 4).

Hence, we found a six-month aortic reintervention-free survival of 50% in the surgical group (efficacy endpoint). Considering the high degree of reinfection (i.e., persistent infection), the aortic complication and reintervention rates described a close monitoring, and frequent follow-up imaging seems mandatory (Table 4). The overall mortality after the two-year mean follow-up was found to be 43% (Figure 3A, Table 4). Regarding the good short- and midterm survival rates of over 90% at six months published lately for both aortic and thoracic VGEIs, surgical graft removal must eventually be considered as “added mortality/morbidity” in this vulnerable cohort [12,13]. Here, the complexity of repair and additional operative procedures had no influence on the in-hospital mortality (Figure 4, Supplementary Table S5). Kahlberg et al. found a trend towards lower 1-year mortality rates when comparing operative and graft-preserving treatments in a systematic review and meta-analysis of 233 cases from 43 studies [17].

The detection of VGEI is most crucial and still a challenge in some cases. Especially regarding the individual operative consequences, sensitivity and specificity must be excellent. Here, the MAGIC criteria showed up to 100% sensitivity, yet were only specific in approx. two-thirds of cases [2,4]. In our cohort, MAGIC positivity was an inclusion criterion; however, most patients “over-fulfilled” the required categories (Supplementary Table S2). ^18^F-FDG PET/CT has been discussed as a method to increase specificity, especially in cases of clinical doubt [5].

To increase diagnostic accuracy, a quantitative approach using focal uptake SUV_max_ and dependent ratios has been introduced; however, studies evaluating VGEI patients and, specifically, comparisons to studies of non-VGEI patients are scarce [7]. Tsuda et al. have suggested an aortic SUV_max_ = 4.5 as a cut-off to diagnose VGEI, while v. Rijsewijk saw a mean SUV_max_ = 9.5 vs. 5.4 in their VGEI-positive vs. VGEI-negative cohort [7,21]. Our analysis identified the SUV_TLR_ with the highest diagnostic accuracy; however, our results showed an aortic SUV_max_ (cut-off 6.45) and an SUV_TBR_ with almost equally high sensitivity and specificity (Figure 2D, Table 2). Along with previous results, the time-to-infection did not influence basic uptake values; however, the type of initial operation (EVAR vs. OAR) might (Table 2) [7,22]. Similar results have been reported from smaller cohort studies and meta-analyses and also after open ascending aortic repair [23,24,25,26,27,28]. Yet, quantitative PET analysis could be suggestive of perigraft gas bubbles, but no correlations with eventual bacteria and clinical outcomes of VGEI are reported [6,29]. Further modalities, such as a white blood cell (WBC) scan, could help shed light on this shortcoming [30]. In our study, no differences regarding the in-hospital mortality were seen (Table 2, Supplementary Table S5). The role of follow-up PET to diagnose persistent infection or reinfection in those patients might help to minimize the high rate of aortic complications during follow-up (Table 4) [31].

While individual case reports suggest isolated removal of PET avid segments, complete removal of the allograft material is common sense in eligible patients according to the ESVS guidelines [3,32]. Here, anatomic reconstruction has proven favorable in terms of infection-free survival [33]. However, despite a clear definition bias, the rate of persistent infection and/or reinfection is high after operative treatment and reached 39.5% in our study (Table 4) [34]. Various groups have published their experiences with specific materials used for reconstruction, mostly for the abdominal aorta, with a lack of direct comparisons. The ESVS guidelines, as well as current meta-analyses, have summarized short-term mortality rates and reinfection rates for biological (pericardium, homograft), prosthetic (silver-coated, biosynthetic) and autologous vein vascular substitutes (VS) [3,16,35]. Generally, mortality rates are almost equal, while resistance to infection might be better for biological and venous VS. Here, we found no significant difference between the three materials used regarding overall mortality and immediate or late aortic complications (Figure 3B, Table 5, Supplementary Tables S5 and S6). However, the study was not designed for this analysis.

The identification of the bacterial/fungal etiology and “evolution/selection” considering the antibiotic regimen applied seems key for a potential targeted therapy and a possible improvement of outcomes for both surgical and conservative treatments given the plethora of microorganisms identified (Supplementary Table S1). Standard culturing along with eventual polymerase chain reaction methods can be of help [12]. However, approx. one-third of blood cultures remain negative, and a polymicrobial flora is frequent [12,17,34]. Hence, preoperative classification according to a single microorganism identified might not be justified [36,37]. The additional sonication of explanted grafts increased the microorganism yield by 16% [38]. Either way, the long-term administration of the correct anti-infective agent along with a possible reevaluation during the post-hospital course is crucial for clinical success and might be even more important than surgical success [3,37,39]. The value of other procedures, i.e., partial graft removal or anti-infective graft irrigation, remains unclear [32,40]. Additionally, patients’ adherence to therapy and possible outpatient parenteral antimicrobial therapy needs to be assessed on a larger scale [41].

Limitations: As is the case with any other study of this kind, our analysis is limited by the heterogeneity of patients, disease and presentation. Generally, the low number of patients prohibits statistical analysis beyond descriptive methods and thus limits the value of our conclusions, specifically for the comparison of conservatively vs. operatively managed patients. Every surgeon might be subjected to an “intention-to-treat” bias for VGEI, or on the other hand, some conservatively managed patients might be frailer than others. Additionally, the presented PET/CT analysis was only available for 53 patients. The mean follow-up was 22.9 ± 26.4 months only, resulting in a calculated follow-up index of 0.75, allowing only short-/midterm conclusions [42].

## 5. Conclusions

VGEI is associated with considerably high in-hospital and short-term complication rates and mortality in this cohort. PET shows excellent diagnostic sensitivity and specificity. The material used for reconstruction might not be crucial for clinical outcomes. With a possible selection bias and insufficient group size, we demonstrate that conservatively managed patients might have a similar outcome. However, a more widespread, register-based approach tailored to a thorough infectious diagnosis and treatment is necessary to identify patients who benefit from this type of surgery, especially during longer follow-ups.

## Figures and Tables

**Table 1:** Patient characteristics, initial operation and VGEI details.

		Combined N = 93	Operative N = 76	Conservative N = 17
**Patient Characteristics**				
age (years; mean ± SD)		65.4 ± 10.6	64.5 ± 10.7	69.6 ± 9.17
sex (male: N; %)		70 (75.3)	55 (72.4)	15 (88.2)
hyperlipidemia		68 (73.1)	55 (72.4)	13 (76.5)
diabetes		28 (30.1)	25 (32.9)	3 (17.6)
nicotine abuse (active)		30 (32.3)	30 (39.5)	0
alcohol abuse (active)		8 (8.6)	8 (10.5)	0
COPD		24 (25.8)	19 (25)	5 (29.4)
renal insufficiency		26 (28)	20 (26.3)	6 (35.5)
dialysis		6 (6.5)	6 (7.9)	0
cancer (history)		21 (22.6)	21 (27.6)	0
arterial hypertension		87 (93.5)	71 (93.4)	16 (94.1)
PAOD		43 (46.2)	37 (48.7)	6 (35.3)
CAD		46 (49.5)	31 (40.8)	15 (88.2)
**Initial Operation**				
EVAR (2xcomplex;1xIBD;1xmono)		29 (31.2)	26 (34.2)	3 (17.6)
TEVAR		11 (11.8)	6 (7.9)	5 (29.4)
OAR		53 (57)	44 (57.9)	9 (52.9)
**VGEI Characteristics**				
early infection		28 (30.1)	22 (28.9)	6 (35.3)
late infection		65 (69.9)	54 (71.1)	11 (64.7)
fistula	cutaneous	8 (8.6)	7 (9.2)	1 (5.9)
	gastrointestinal	21 (22.6)	17 (22.4)	4 (23.5)
	ureter	3 (3.2)	3 (3.9)	-
B-symptoms		48 (51.6)	37 (48.7)	11 (64.7)
lab	leucocytes (cells/µL)	15.9 ± 6.7	11.2 ± 5.1	10.1 ± 4.5
	CRP (mg/dL)	11.9 ± 12.7	11.8 ± 13.2	12.3 ± 10.7
	PCT (ng/mL)	2.6 ± 9.0	2.8 ± 9.6	1.2 ± 1.6
preOP CT drain		25 (26.9)	18 (23.7)	7 (41.2)

COPD = chronic obstructive pulmonary disease; PAOD = peripheral arterial occlusive disease; CAD = coronary artery disease; complex = complex EVAR with fenestrations/scallop; IBD = iliac branch device; mono = mono-iliac EVAR with crossover bypass; normal range: leucocytes (3.8–9.8 cells/µL); C-reactive protein (CRP) (<5.0 mg/dL); procalcitonin (PCT) (<0.5 ηg/mL).

**Table 2:** Quantitative intensity uptake analysis from PETs comparing VGEI patients with a non-VGEI control cohort.

Quantitative/Qualitative Analysis		VGEI + PET N = 53	Control Group N = 19	*p*
**SUV_max_ aorta**		12.5 ± 7.3	4.2 ± 1.7	**<0.001**
		6.45 at 83% sensitivity/89% specificity		
SUV_TLR_ (target-to-liver ratio)		3.6 ± 2.0	1.2 ± 0.4	**<0.001**
		1.35 at 94% sensitivity/89% specificity		
SUV_TBR_ (target-to-back ratio)		4.8 ± 3.1	1.4 ± 0.4	**0.001**
		2.25 at 89% sensitivity/94% specificity		
SUV_max_ liver		3.6 ± 0.9	3.7 ± 0.9	0.63
SUV_max_ blood pool		2.7 ± 0.7	3.0 ± 0.5	0.21
Visual grading scale (VGS)	0	-	1 (5.3)	**<0.001**
	1	-	-	
	2	-	14 (73.7)	
	3	6 (11.3)	4 (21.1)	
	4	47 (88.7)	-	
**infection time**		early infection 15 (28.3)	late infection 38 (71.7%)	
SUV_max_ aorta		12.27 ± 5.59	12.78 ± 7.95	0.94
SUV_TLR_		3.55 ± 1.76	3.68 ± 2.18	0.9
SUV_TBR_		4.41 ± 2.09	3.5 ± 4.78	0.89
**fistula**		fistula 19 (36.5%)	no fistula 33 (63.5%)	
SUV_max_ aorta		13.54 ± 8.04	11.06 ± 5.92	0.31
SUV_TLR_		3.79 ± 2.25	3.37 ± 1.68	0.61
SUV_TBR_		5.1 ± 3.67	4.21 ± 1.91	0.54
**initial treatment**		endovascular 26 (50%)	open 26 (50%)	
SUV_max_ aorta		14.63 ± 8.66	10.64 ± 5.27	0.08
SUV_TLR_		4.25 ± 4.42	3.02 ± 1.38	0.07
SUV_TBR_		5.82 ± 3.95	3.37 ±1.53	**0.03**
**safety EP reached**		in-hospital survivor 45 (84.9)	in-hospital death 8 (15.1)	
SUV_max_ aorta		11.90 ± 7.35	16.0 ± 5.77	0.06
SUV_TLR_		3.54 ± 2.14	3.89 ± 1.36	0.24
SUV_TBR_		4.71 ± 3.23	5.13 ± 2.30	0.36

SUV = standard uptake value. Data shown as mean standard deviation (upper line) and cut-off value from Youden analysis with the respective highest sensitivity and specificity (lower line) (Figure 2D). Comparison of SUV values for infection time, presence of fistula, type of initial treatment and safety endpoint reached (Mann–Whitney U test for comparison, *p* < 0.05 is considered significant and highlighted bold).

**Table 3:** VGEI replacement operative details.

		**N = 76**
**operative setting**		
emergency operation		6 (6.5)
extent	thoracic	8 (10.5)
	thoraco-abdominal	4 (5.3)
	abdominal	64 (84.2)
fistula	cutaneous	7 (9.2)
	gastrointestinal	17 (22.4)
	ureter	3 (3.9)
**procedural details**		
operating time (min)		61.3%
transfusion (# RBC concentrates)		9 (9–12)
reco	tube	19 (25.3)
	bifurcation	56 (74.7)
material	pericardium	46 (61.3)
	silver-coated Dacron	16 (21.3)
	deep vein	13 (17.3)
renal cold perfusion		4 (5.3)
ECMO (partial left heart bypass)		13 (17.1)
omentum plasty		8 (10.5)
gastrointestinal resection		12 (15.8)
gastrointestinal direct suture		4 (5.3)
pulmonary resection/suture		2 (2.6)
other		13 (17.1)
**postoperative course (in-hospital)**		
scheduled revision		6 (7.9)
additional drainage (CT)		28 (36.8)
complication rates	aortic	18 (23.7)
	bleeding/rupture	16 (21.0)
	neurologic	8 (10.5)
	stroke	3 (3.9)
	surgical	47 (61.8)
	SSI	22 (28.9)
	limb ischemia	10 (13.2)
	visceral complication	9 (11.8)
	medical	50 (65.8)
	acute kidney failure	19 (25)
	dialysis (temp)	10 (13.2)
	respiratory problems	15 (19.7)
	pulmonary embolism	2 (2.6)
	myocardial infarction	4 (5.3)

Two patients were considered urgent (no emergency). One operation was discontinued after laparotomy due to unexpected inoperability. ECMO = extracorporeal membrane oxygenation: used as a partial left heart bypass; RBC = red blood cell; SSI = surgical site infection; other additional procedures included 4× splenectomy; 1× partial vertebral body resection; 2× cholecystectomy, 2× sartorius flap; 1× nephrectomy; 1× sublay mesh augmentation; 1× psoas hitch plasty; and 1× partial ureterectomy.

**Table 4:** Outcome analysis by treatment.

		Combined N = 93	Operative N = 76	Conservative N = 17	
outcome analysis					
days in hospital		45 ± 37	51 ± 38	21 ± 15	
days in ICU		17 ± 34	20 ± 37	3 ± 7	
surgical	in-hospital complication (rate)	-	47 (61.8)	-	
medical			50 (65.8)		
neurologic			8 (10.5)		
aortic			18 (23.7)		
in-hospital	aortic reintervention (rate)	-	13 (17.1)	-	
6 months			14 (18.4)		
12 months			21 (27.6)		
overall			23 (30.3)		
30 d	mortality (rate)	11 (11.8)	8 (10.5)	3 (17.6)	
**in-hospital**		18 (19.4)	15 (19.7)	3 (17.6)	
6 months		28 (30.1)	24 (31.5)	4 (23.5)	
12 months		32 (34.3)	30 (39.5)	4 (23.5)	
overall		40 (43)	35 (46.1)	5 (29.4)	
**6 months aortic reintervention-free survival**		-	38 (50)	-	
reinfection (persistent)		-	30 (39.5)	17 (100)	-
follow-up (months)		22.9 ± 26.4	24.1 ± 24.2	17.9 ± 35	

ICU = intensive care unit. The safety and efficiency endpoints are highlighted in bold.

**Table 5:** Abdominal replacement material analysis.

N = 64 Abdominal Replacement	Pericardium N = 38	Silver-Coated Dacron N = 13	Femoral Vein N = 13	*p*
aortic complication (in-hospital)	10 (26.3)	1 (7.7)	4 (30.8)	0.31
anastomotic bleeding	7 (18.4)	1 (7.7)	3 (23.1)	0.64
fluid collection (drainage)	2 (5.3)	-	1 (7.7)	-
anastomotic stenosis	1 (2.6)	-	-	
surgical complication	21 (55.3)	10 (76.9)	11 (84.6)	0.09
impaired wound healing	8 (21.1)	5 (38.5)	7 (53.8)	0.07
aortic complication during FU	12 (31.6)	4 (30.8)	5 (38.5)	0.89
death in-hospital	8 (21.1)	0	3 (23.1)	0.18
death during FU	18 (47.4)	6 (46.2)	4 (30.8)	0.57
(persistent) reinfection	17 (44.7)	7 (53.8)	3 (23.1)	0.25

A direct comparison of relevant outcome parameters regarding the replacement material used in the abdominal cohort. Chi-Square test for comparison; <0.05 is considered significant and highlighted bold.

## Data Availability

All original data can be obtained from the corresponding author upon request.

## Supplementary Materials

The following supporting information can be downloaded at https://www.mdpi.com/article/10.3390/jcm13010269/s1; Figure S1: Visual Grading Scale VGS PET/CT analysis. Figure S2: Univariate analysis re-(persistent)-infection. Table S1: Microbiology data for individual patients. Table S2: MAGIC criteria. Table S3: Comparison PET/CT VGEI and control cohort characteristics. Table S4: Complication rates operative cohort after Clavien-Dindo. Table S5: Univariate analysis of in-hospital mortality. Table S6: Univariate analysis of re-infection (persistent).
