# Supplementary material for: Aortic Vascular Graft and Endograft Infection–Patient Outcome Cannot Be Determined Based on Pre-Operative Characteristics
Source: J Clin Med. 2024 Jan 3;13(1):269. doi: 10.3390/jcm13010269 (PMC10779700; doi:10.3390/jcm13010269)
Supplement: Supplementary file 1 [file jcm-13-00269-s001.zip › Supplementary Tables.pdf]

Table S1. Microbiology data for individual patients. The initial graft and the time to infection is shown. One patient (52) had a vena cava fistula. *neg* = blood culture/intraOP swab negative; empty cell = non taken/retrieved; *cons* = conservative treatment; <u>Bacteria:</u> a = E. faecium; b = K. pneumoniae; c = E. coli; d = C. albicans; e = Strept. sanguinis; f = Staph. epidermidis; g = NEST; h = MRSA; i = Strept. dysgalactiae; j = S. marcescens; k = Salmonella enterica; m = Staph. capitis; n = VRE Vancomycin resistant Enterococcus; o = Corynebact. spec.; p = Staph. aureus; q = Prot. vulgaris; r = Bact. species; s = E. faecalis; t = C. glabrata; u = Staph. haemolyticus; v = Clostridium spec.; w = 3MRGN; y = Citrobacter freundii; z = Strept. anginosus; $ = Strept. mitis; & = Cutibacterium acnes; § = P. aeruginosa; ß = Propionibact. acnes; α = Enterococcus avium; δ = Lactobacillus plantarum; ε = Candida parapsilosis; ζ = Listeria monocytogenes; η = Staph. warneri; θ = Finegoldia magna; λ = Streptococcus oralis; μ = Enterobacter cloacae; ξ = Veiilonella parvula; π = Staph. hominis; σ = Streptococcus agalactiae; φ = Proteus mirabilis; ψ = Streptococcus intermedius; ω = Streptococcus constellatus; ϐ = Egerthella lenta; ϑ = Solobacterium Moorei; ϕ =Lactobacillus rhamnosus; ϖ = Klebs. aerogenes; Ϛ = Eikinella spp; Ϫ = Atopium parvalum; э = Actinomyces odontolyticus; SUV = standard uptake value; VGS = visual grading scale; outcome: x = respectice endpoint (EP) reached.

#	graft			time to VGEI (mo)	fistula				microbiology							PET				out come	
	EVAR	TEVAR	OR		cutaneous	esophagus	duodenum	ureter	blood culture	Δ time (d)	biopsy	Δ time (d)	swab	Δ time (d)	swab @ OP	SUV_{max} aorta	SUV_{max} liver	SUV_{max} blood	VGS (0-4)	safety EP	efficacy EP
1	X			48	X				*neg*	1					k	16.4	2.6	2.1	4	X	X
2			X	155							a	189	m	4	b,n	4.7	3.3	1.8	4	X	-
3			X	2					d	17										X	-
4		X		30		X			*neg*	64					d,o	22.2	6	4	4	-	-
5			X	1											c					X	-
6			X	76	X								f	64	f					X	X
7			X	62	X				*neg*	1					d	24.3	3.5	3	3	X	X
8			X	92					p	1					p					X	X
9	X			7					c	2					c					X	-
10			X	1											c,q,r,s					X	-
11			X	1					*neg*	17			p,s	10	b					X	X
12			X	78			X		*neg*	27			b,c	5	a,b,c,d,t	4.6	2.6	2.1	3	X	-
13			X	24	X										c,d	8.7	2.7	2.6	4	X	-
14			X	40											a,u					-	-
15	X			32			X								f	8.2	3.2	3.4	4	X	-
16			X	1					*neg*	4					f					-	-
17	X			25			X								v					-	-

18			X	1					*neg*	17					c					-	-
19			X	121			X		*neg*	9					b,w	12.2	3.5	3.1	4	X	X
20		X		4					f	99					s,u	18.3	4.5	4	4	X	-
21	X			35					*neg*	2					w,y	8.9	3.4	3	4	X	-
22			X	123					*neg*	0					z					X	X
23	X			29					i	10					u					X	X
24	X			102					*neg*	1					*neg*	22.2	6	4	4	-	-
25			X	37			X								y					X	X
26			X	1	X				*neg*	18	h	16			*neg*					X	X
27			X	156	X				*neg*	9					f	11.3	3.6	2.8	4	X	X
28			X	160					*neg*	4					d					X	X
29		X		24		X			*neg*	1					z,$					-	-
30	X			2					*neg*	5					*neg*	13.4	3.8	4.2	4	-	-
31	X			4											j	16.3	2.4	1.6	4	-	-
32			X	5											*neg*					-	-
33	X			3			X		k	1					k	18.5	3.2	3.1	4	X	X
34	X			24					*neg*	11					c	25.9	4.7	3.6	6	X	X
35	X			16			X		*neg*	2					$					X	-
36	X			8					*neg*	9					d	13.4	3.3	2.6	4	-	-
37	X			1					*neg*	1					*neg*	22.2	5.4	4.1	4	-	-
38	X			14					*neg*	22	p	12			*neg*					X	X
39			X	1					u	3					c					X	-
40			X	65					*neg*	1	*neg*	12			&	6.4	5.4	-	4	X	-
41	X			1											d	13.1	2.4	2	4	X	-
42	X			11					§	13	§	7			§	19.1	3.5	2.3	4	X	X
43	X			6					f	3					f	6.7	2.9	2.9	4	X	X
44		X	X	132											ß	15	4	2.8	4	X	X
45		X		0					*neg*	16	o	15	p	15	o					X	-
46			X	36				X							f	8.4	2.4	2.5	4	X	-
47		X		3					*neg*	6	*neg*	7			*neg*					X	X
48			X	133				X	*neg*	2					*neg*					X	-
49			X	209			X		*neg*	2					d,α,δ	10.6	4.2	2.9	4	X	-
50			X	40											a					X	-
51			X	79			X		f	14					*neg*	9.9	3.5	3.2	3	X	-
52	X			0					*neg*	7					s	5.5	3.5	1.9	4	X	-
53	X			1					*neg*	6			h,ε	21	h,§	4.5	3.2	2.7	3	-	-
54	X			0					*neg*	15					*neg*	12.8	2.8	1.6	4	X	-
55			X	14											f					X	-
56			X	15					ζ	14	ζ	17				4	2.7	2.5	4	X	-
57			X	52	X				u,η	26	u,&,η,θ	12			*neg*	3.4	3.3	2.2	3	X	X
58		X		28		X			*neg*	20	ξ,μ,λ	19			a,π,μ,d	5.1	2.3	1.4	4	X	-
59			X	3					a,s	36			s,§	6	s,§	8.9	3.4	2.2	4	X	X
60	X			95					*neg*	14	p	26				5.9	4.4	2.1	4	X	X
61	X			42					*neg*	0					σ					X	X
62	X			29					c	7	c,s,d,§,φ	12			a,c,s,d,§,φ,r					X	-
63			X	107			X		*neg*	3					c,s,ψ,ω					X	-
64			X	0					*neg*	12			a,d	9	a,d,ϐ					X	-
65	X			58					*neg*	8					b,p,q,s,z,ϑ					X	-
66			X	26			X		*neg*	13	c,ϕ	14			c,d,ϕ	7.7	2.8	1.6	4	X	X
67			X	25					z	9	c,r,z	9			*neg*					X	-
68			X	20	X			X			c	2			*neg*	6.8	2.6	2.2	4	X	-
69			X	14					*neg*	13			a,ϖ	10	a					-	-
70			X	6			X		*neg*	0					Ϛ					X	-
71			X	34					*neg*	10					s	13.8	3.6	3.2	4	-	-
72			X	6		X			*neg*	6	z,$,ω	5			z,$,ω,Ϫ					X	X
73		X		0					f	38	f	33			μ	4.4	3.4	2.9	4	X	-

74			X	11			X		*neg*	2					z,ф,э					X	X
75	X			3					*neg*	7	c	7			c	17.5	3.1	2.7	4	X	X
76			X	2											f	17.9	3.4	2.9	4	X	X
77	X			66	X						a				*cons*	16.3	2.7	2.2	4	X	
78	X			40					b						*cons*	9.8	3.3	2.2	4	X	
79		X		1		X					c,d				*cons*	10	4.6	3.1	4	X	
80		X		77					e						*cons*	44	3.6	2.1	4	X	
81		X		10		X					a,f				*cons*					-	
82		X		1		X			*neg*						*cons*					X	
83			X	2							d				*cons*					-	
84	X			65		X			g						*cons*					-	
85			X	2							b				*cons*	6.9	3.2	2.8	4	X	
86			X	147											*cons*	11.6	3.1	2.9	4	X	
87			X	1					f						*cons*	20.5	3.6	3.3	4	X	
88			X	60											*cons*	7.9	4.9	3.3	3	X	
89			X	180					h		h				*cons*	8.8	3.1	2.3	4	X	
90			X	53					c						*cons*	7.5	4.1	3.5	4	X	
91	X	X		12					f						*cons*	18	2.5	1.9	4	X	
92			X	60					d,j						*cons*	15	5.3	3.8	4	X	
93			X	1					*neg*						*cons*	7.9	4.3	3.4	4	X	

Table S2. MAGIC criteria. Given is the absolute number (percentage) of patients with positive major and/or minor criteria in three categories. Chi-Sqare test to compare operative vs. conservative in the respective category, $p<0.05$ is considered significant and highlighted bold.

	**Lyons criteria**					
	surgical/clinical		radiologic		laboratory	
	major	minor	major	minor	major	minor
**combined (N=93)**	56 (60.2)	53 (57.0)	77 (82.8)	37 (39.8)	83 (89.2)	66 (71.0)
operative (N=76)	46 (60.5)	43 (56.6)	29 (38.2)	62 (81.6)	58 (76.3)	68 (89.5)
conservative(N=17)	8 (47.1)	12 (70.6)	8 (47.1)	15 (88.2)	8 (47.1)	15 (88.2)
p	0.16		0.68		0.06	

Table S3. Comparison PET/CT VGEI and control cohort characteristics. Age = age at initial operation; patient characteristics missing in four control group patients; Chi-Sqare test to compare VGEI+PET cohort vs. control group and VGEI+PET cohort vs. entire VGEI cohort, respectively; p<0.05 is considered significant and highlighted bold.

	VGEI+PET N=53	control group N=19	p	p vs. combined (**Table I**)
**patient characteristics**				
age (years; mean ± SD)	64.8 ± 10.7	68.5 ± 8.9	0.48	0.8
sex (male: N; %)	40 (75.5)	17 (89.4)	0.19	0.98
hyperlipidemia	39 (73.6)	12 (70.6)	0.39	0.91
diabetes	15 (28.3)	4 (23.5)	0.54	0.82
nicotine abuse (active)	16 (30.2)	1 (5.3)	0.06	0.66
alcohol abuse (active)	6 (11.3)	0	0.19	0.46
COPD	13 (24.5)	4 (23..5)	0.76	0.81
renal insufficiency	19 (35.8)	7 (41.2)	0.94	0.06
dialysis	3 (5.7)	0	0.56	0.72
cancer (history/present in control)	10 (18.9)	19 (100)	**<0.001**	0.45
arterial hypertension	49 (92.5)	14 (82.4)	**0.04**	0.7
PAOD	23 (43.4)	0	**<0.001**	0.53
CAD	28 (52.8)	8 (47.1)	0.59	0.54
**initial operation**				
EVAR	20 (37.7) (1xcomplex;2xmono)	15 (78.9) (1x complex)	**0.02**	0.32
TEVAR	7 (13.2)	2 (10.5)		
OAR	26 (49.1)	2 (10.5)		
rupture	9 (17.0)	2 (10.5)	0.72	0.79
ex domo	28 (54.7)	9 (47.4)	0.68	0.68
**VGEI characteristics**				
time-to-infection (months)	45.4 ± 52.3			0.2
early infection	15 (28.3)			0.73
late infection	38 (71.7)			
fistula: cutaneous	6 (11.3)			0.46
fistula: gastro-intestinal	10 (18.9)			0.45
fistula: ureter	2 (3.8)			0.73
B-symptoms	30 (56.6)			0.27
Lab: leucocytes (cells/µL)	9.9 ± 3.6			0.07
Lab: CRP (mg/dL)	9.8 ± 8.7			0.53
Lab: PCT(ng/ml)	3.9 ± 12.6			0.33
**treatment strategy**				
operative (vs. conservative)	40 (75.5)			

Table S4. Complication rates operative cohort after Clavien-Dindo.

		**combined** N = 76
**postoperative course**		
complication rates Clavien-Dindo	type I	5 (6.6)
	type II	21 (27.6)
	type IIIa	6 (7.9)
	type IIIb	16 (21.1)
	type Iva	9 (11.8)
	type IVb	1 (1.3)
	type V	15 (19.7)

Table S5. Univariate analysis of in-hospital mortality. Logistic regression analysis; p<0.05 is considered significant and highlighted bold.

in-hospital mortality		Odds ratio	95%-confidence interval	p
**patient characteristics**				
sex (male vs. female)		0.84	0.25 – 2.87	0.78
age (+1year)		**1.06**	**1.01 – 1.12**	**0.04**
+1 co-morbidity		1.19	0.87 – 1.63	0.27
diabetes		1.63	0.56 – 4.78	0.37
nicotine abuse (active)		0.36	0.09 – 1.34	0.13
COPD		1.58	0.52 – 4.82	0.42
renal insufficiency		1.38	0.45 – 4.15	0.57
dialysis		4.8	0.88 – 26.12	0.07
cancer (history)		1.42	0.44 – 4.57	0.56
PAOD		1.21	0.43 – 3.38	0.72
CAD		1.8	0.63 – 5.14	0.28
**initial operation**				
aortic aneurysm		**4.76**	**1.02 – 22.3**	**0.047**
occlusive disease		0.28	0.06 – 1.33	0.11
aortic dissection		-	-	-
EVAR		1.93	0.67 – 5.53	0.22
TEVAR		1.3	0.32 – 5.31	0.72
OAR		0.42	0.15 – 1.22	0.11
rupture		2	0.61 – 6.72	0.25
**VGEI characteristics**				
early infection		0.78	0.45 – 1.38	0.41
late infection		1.53	0.47 – 4.97	0.48
fistula		0.65	0.21 – 2	0.45
B-symptoms		**4.22**	**1.27 – 14**	**0.02**
**PET quantitative analysis**				
SUV_{max} aorta (+4)		1.29	0.89 – 1.87	0.17
SUV_{TLR} (+1)		1.08	0.77 – 1.52	0.66
SUV_{TBR} (+1)		1.04	0.84 – 1.29	0.72
**operative setting**				
emergency operation		1.01	0.26 – 4	0.99
extent	thoracic	0.33	0.03 – 4.19	0.31
	thorac-abdominal	0.21	0.03 – 1.64	0.14
	abdominal	1.54	0.35 – 6.8	0.56
**procedural details**				
operating time (+30min)		0.96	0.86 – 1.07	0.48
reco	tube	**5.2**	**1.56 – 17.36**	**0.007**
	bifurcation	**0.28**	**0.09 – 0.93**	**0.04**
material	pericardium	3.17	0.81 – 12.41	0.1
	silver coated Dac	-	-	-
	deep vein	1.28	0.31 – 5.35	0.74
renal cold perfusion		4.54	0.58 – 35.25	0.15
ECMO		2.1	0.55 – 8.07	0.28
omentum plasty		2.8	0.59 – 13.34	0.19
gastro-intestinal resection		0.33	0,04 – 2.74	0.31
gastro-intestinal direct suture		4.54	0.58 – 35.25	0.15
**postoperative course**				
aortic complication		1.85	0.54 – 6.36	0.33
neurologic complication		0.55	0.06 – 4.86	0.59
surgical complication		0.65	0.21 – 2.1	0.45
medical complication		2.4	0.62 – 9.5	0.21
**outcome parameters**				
re-infection (persistent)		0.56	0.2 – 1.59	0.28

Table S6. Univariate analysis of re-infection (persistent). Cox regression analysis; $p<0.05$ is considered significant and highlighted bold.

**re-infection (persistence)**		Hazard ratio	95%-confidence interval	p
**patient characteristics**				
sex (male vs. female)		1.36	0.63 – 2.98	0.62
age (+1year)		1.03	1 – 1.06	0.06
+1 co-morbidity		0.99	0.83 – 1.19	0.97
diabetes		1.38	0.64 – 2.96	0.41
nicotine abuse (active)		0.97	0.46 – 2.1	0.94
COPD		1.8	0.79 – 3.91	0.17
renal insufficiency		0.68	0.27 – 1.69	0.42
dialysis		1.78	0.53 – 5.96	0.35
cancer (history)		1.2	0.51 – 2.9	0.67
PAOD		1.08	0.53 – 2.26	0.82
CAD		1.02	0.47 – 2.22	0.95
**initial operation**				
aortic aneurysm		0.95	0.45 – 2.01	0.89
occlusive disease		1.04	0.48 – 2.24	0.92
aortic dissection		1.11	0.15 – 8.23	0.92
EVAR		0.91	0.4 – 2.1	0.82
TEVAR		0.71	0.17 – 2.97	0.63
OAR		1.39	0.63 – 3.08	0.41
rupture		0.98	0.34 – 2.84	0.97
ex domo		1.55	0.71 – 3.35	0.27
**VGEI characteristics**				
early infection		1.11	0.72 – 1.72	0.63
late infection		2.01	0.56 – 7.28	0.28
fistula		1.01	0.48 – 2.1	0.98
B-symptoms		1.4	0.67 – 3.07	0.34
**operative setting**				
emergency operation		**2.41**	**1.07 – 5.46**	**0.035**
extent	thoracic	1.29	0.47 – 3.56	0.62
	thorac-abdominal	0.71	0.63 – 7.79	0.77
	abdominal	1.12	0.15 – 8.3	0.91
**procedural details**				
operating time (+30min)		0.97	0.91 – 1.04	0.43
reco	tube	0.85	0.32 – 2.24	0.74
	bifurcation	0.92	0.37 – 2.27	0.85
material	pericardium	0.94	0.57 – 1.56	0.81
	silver coated Dac	2.22	0.59 – 8.39	0.53
	deep vein	1.49	0.43 – 5.1	0.24
renal cold perfusion		-	-	-
ECMO		1.25	0.47 – 3.28	0.66
omentum plasty		1.56	0.47 – 5.2	0.47
gastro-intestinal resection		1.43	0.61 – 3.37	0.42
gastro-intestinal direct suture		1.9	0.47 – 8.47	0.35
pulmonary resection/suture		-	-	-
**postoperative course**				
aortic complication		1.48	0.65 – 3.39	0.35
neurologic complication		0.54	0.12 – 2.23	0.4
surgical complication		1.78	0.78 – 4.06	0.17
visceral complication		**4.5**	**1.7 – 11.87**	**0.002**
medical complication		1.12	0.52 – 2.42	0.77
