# Supplementary figures and images for: Aortic Vascular Graft and Endograft Infection–Patient Outcome Cannot Be Determined Based on Pre-Operative Characteristics
Source: J Clin Med. 2024 Jan 3;13(1):269. doi: 10.3390/jcm13010269 (PMC10779700; doi:10.3390/jcm13010269)

VGS 0 – no uptake

VGS 1

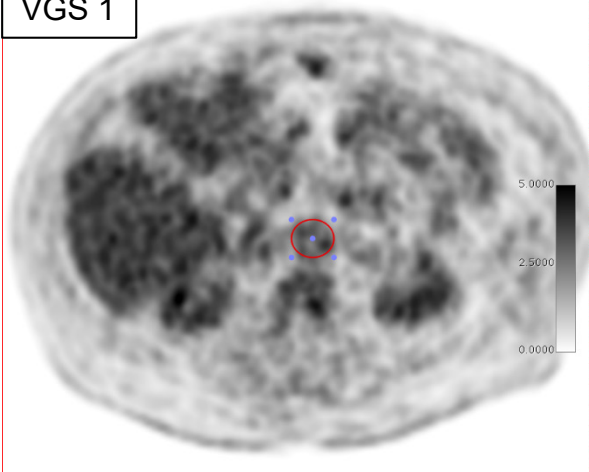

VGS 2

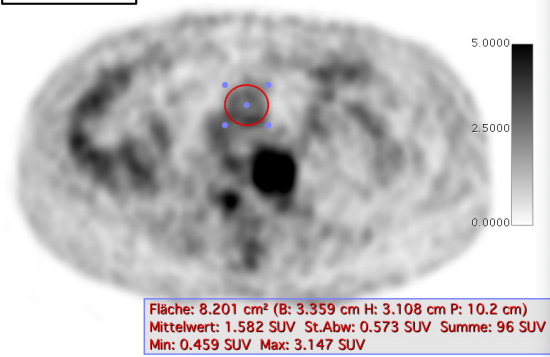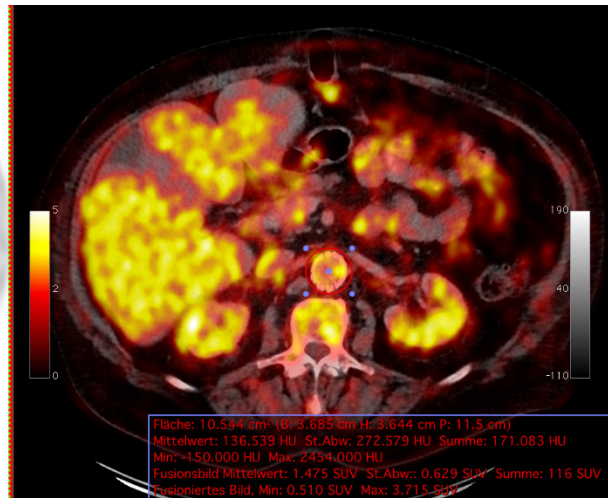

VGS 3

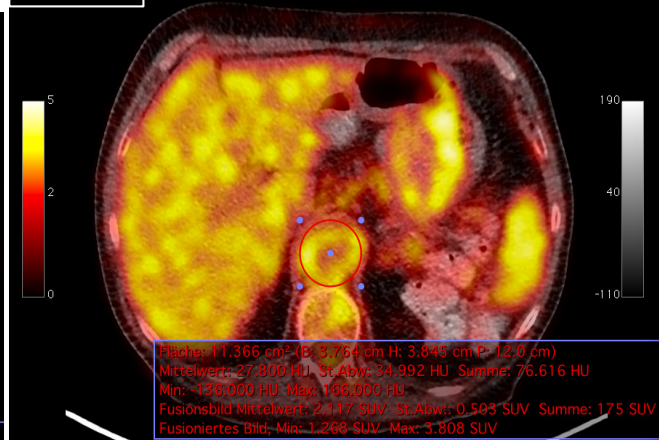

VGS 4

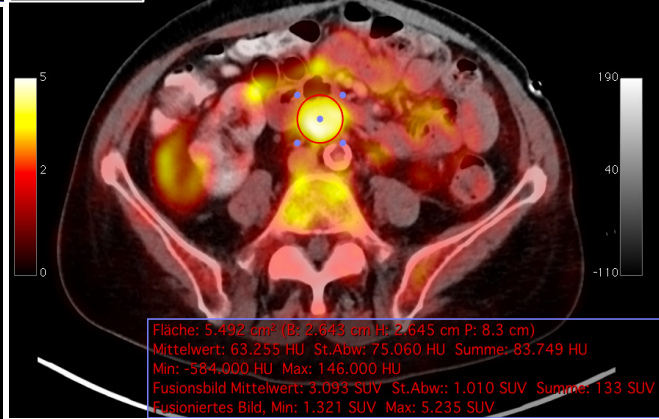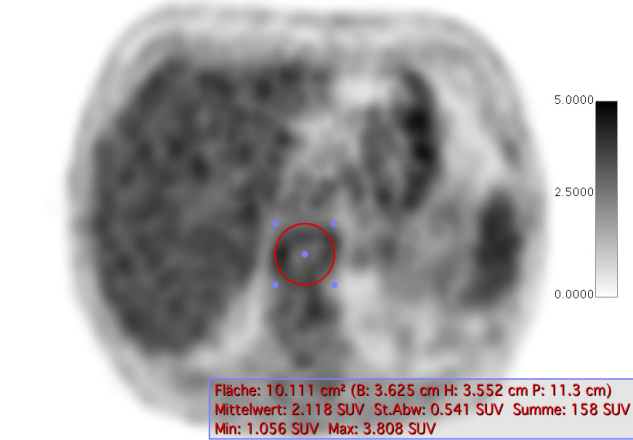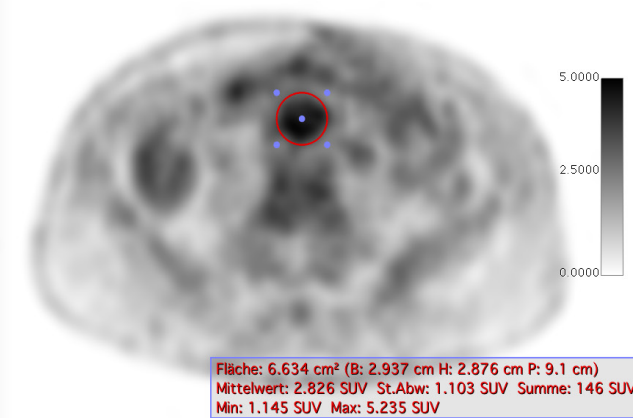

Supplement: Supplementary file 1 [file jcm-13-00269-s001.zip › Supplementary Figure S1.pdf]

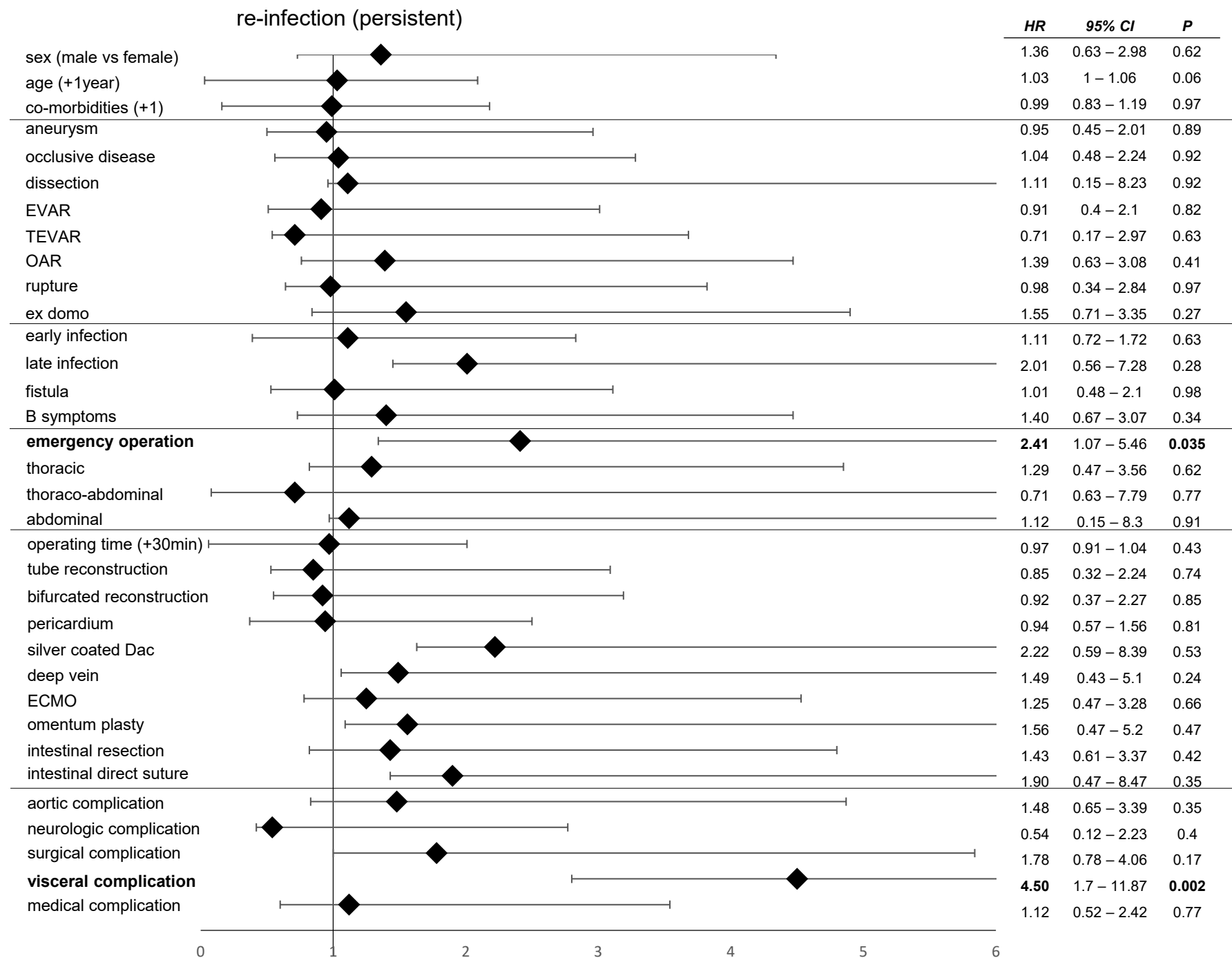

Supplement: Supplementary file 1 [file jcm-13-00269-s001.zip › Supplementary Figure S2.pdf]
